# Barriers to access cancer screening and treatment services in Germany

**DOI:** 10.1192/j.eurpsy.2022.561

**Published:** 2022-09-01

**Authors:** F. Baessler, A. Zafar

**Affiliations:** Centre for Psychosocial Medicine, Department Of General Internal And Psychosomatic Medicine, Heidelberg, Germany

**Keywords:** oncology, psychiatry, mental healthcare, masculinity

## Abstract

**Introduction:**

individual attitudes and structural inadequacies act as major barriers towards non-utilization of cancer screening and treatment offers in many high-income countries with subsidized public healthcare.

**Objectives:**

Our interdisciplinary research group at Heidelberg University is studying the underlying individual perceptions, attitudes and experiences of age- and gender-specific barriers against cancer-related medical and psychosocial offers available in Germany.

**Methods:**

We designed a mixed-methods, sequential explanatory study using two quantitative instruments to determine the most important age- and gender-specific barriers for non-patients and cancer patients and survivors. In the second phase, semi-structured interviews will be conducted via selective sampling to record participant opinions, experiences and expectations of using cancer-related health services.

**Results:**

We expect to identify and explain important personal barriers and facilitators related to the use of cancer screening and treatment offers. Further interviews with stakeholders in cancer healthcare, such as physicians, nurses and self-help groups will be conducted to complement data from the service-providers’ point of view. The results will be analyzed with behavioral and sociocultural theories to gain a deeper understanding of perceived and experienced barriers in accessing cancer care in Germany and to formulate recommendations for prospective targeted approaches and interventions.

**Conclusions:**

Our findings will be useful for facilitating knowledge transfer and policy dissemination to increase public awareness about cancer offers and improve participation rates. The results will be also used to develop an interprofessional teaching module in the medical curriculum as well as prepare and implement advanced training courses for medical professionals certified by the State Medical Association.

**Disclosure:**

This study is funded by Strube Stiftung Stuttgart.

